# Skin substitutes are more potent than dermal or epidermal substitutes in stimulating endothelial cell sprouting

**DOI:** 10.1186/s42490-019-0018-8

**Published:** 2019-07-17

**Authors:** Hanneke N. Monsuur, Ester M. Weijers, Susan Gibbs, Lenie J. van den Broek

**Affiliations:** 1 Department of Molecular Cell Biology and Immunology, Amsterdam UMC, Vrije Universiteit Amsterdam, Amsterdam, The Netherlands; 2 Department of Oral Cell Biology, Academic Center for Dentistry Amsterdam (ACTA), University of Amsterdan and Vrije Universiteit Amsterdam, Amsterdam, The Netherlands

**Keywords:** Skin substitute, Angiogenesis, Wound healing, Endothelial cell, Chronic wounds

## Abstract

**Background:**

Therapy resistant ulcers are wounds that remain open for a long time period and often arise from chronic venous disease, prolonged pressure or diabetes. For healing of chronic wounds, revitalization of the inert wound bed, which is achieved by angiogenic sprouting of new blood vessels is of great importance. An alternative treatment option to conventional therapies is the use of skin substitutes: dermal (DS), epidermal (ES) or bi-layered skin substitutes (SS). The aim of this study was to determine the mode of action of an autologous SS, ES and DS with regards to endothelial cell proliferation, migration and angiogenic sprouting into a fibrin hydrogel.

**Results:**

SS consists of a fully differentiated epidermis expanding over the acellular donor dermis (AD) which has become repopulated with fibroblasts. DS is the same construct as SS but without the epidermis and ES is the same construct as SS but without the fibroblasts. As a control, AD was used throughout. It was found that the bi-layered SS was the most potent substitute in inducing migration and sprouting of endothelial cells. The cross talk between dermis and epidermis resulted in the strongest induction of sprouting via VEGF and uPAR. ES stimulated sprouting more than DS again via VEGF and uPAR. The slight induction of sprouting mediated by DS was not mediated by VEGF, but was in part stimulated through uPAR.

**Conclusion:**

This in vitro study supports our clinical observations that a bi-layered SS is a strong stimulator of angiogenesis and therefore has the potential to revitalize an inert wound bed.

## Background

Therapy resistant ulcers are wounds that remain open for a long time period, showing no signs of improvement within 3 months of optimal care [1]. Ulcers often arise from chronic venous disease, prolonged pressure or diabetes, are difficult to treat and show a high rate of recurrence [2–4]. They greatly influence the quality of life of patients who suffer from prolonged pain, social isolation and depression [5]. Since therapy resistant ulcers affect approximately 1–2% of the population, they form a large financial burden to society [6]. Also, it is thought that the prevalence will only rise further due to the increasing age of the population and increased prevalence of underlying diseases like diabetes and vascular disease [3].

Several treatment options are available, such as compression therapy, infection control, wound bed debridement, dressings, surgery and adjuvant agents [7]. Despite many treatment options the recurrence rate of chronic ulcers is up to 70% [7]. An alternative treatment option is the use of skin substitutes; either acellular, dermal (DS), epidermal (ES) or bi-layered skin substitutes (SS) (reviewed by [8, 9]). Notably, SS consisting of a reconstructed epidermis on a fibroblast-populated dermis are showing promising results in clinical studies and one (Apligraf® from Organogenesis, Massachusetts, USA) is now FDA approved and commercially available. The use of dermal or epidermal substitutes are less frequently reported for treating ulcers.

Previously, we have described an autologous SS for treating hard-to-heal chronic wounds [1, 10]. The skin substitute is made from 3 mm punch biopsies obtained from the patient to be treated and consists of a reconstructed epidermis on a fibroblast populated (acellular) donor dermis [10]. In a retrospective study 66 ulcers ((arterio)venous, decubitus, or post-operative) were treated with a single application of the skin substitute. After 24 weeks complete closure was observed in 55% of the ulcers and an additional 29% of the ulcers showed 50–99% closure. The ulcers that completely closed showed a recurrence rate of only 16% 1 year after closure [1]. The mode of action of this SS is thought to be in its ability to revitalize the inert non healing wound bed by stimulating granulation tissue formation. Indeed granulation tissue in the ulcer wound bed is regarded as an indicator of ulcer healing, while poor granulation tissue formation is a feature of non-healing chronic wounds [11]. Granulation tissue consists of a provisional extracellular matrix, wound healing factors and blood vessels formed by fibroblasts and endothelial cells entering the wound bed. It has been shown that SS secrete a more potent cocktail of wound healing factors than DS (fibroblasts only) or ES (keratinocytes only) due to synergistic paracrine feedback mechanisms occurring between the fibroblasts and keratinocytes in the skin substitute [12]. In this study, we further compared the mode of action of SS, ES and DS with regards to stimulating angiogenesis. The influence of the different skin substitutes on endothelial cell proliferation, migration and angiogenic sprouting was investigated. Vascular endothelial growth factor is a potent chemoattractant for angiogenesis and the urokinase plasminogen activator receptor (uPAR) enhances pericellular proteolysis by serving as a docking site to uPA which in turn triggers a cascade of proteolytic events that lead to the active degradation of extracellular matrix thus facilitating vessel invasion into the extracellular matrix [13]. Therefore, it was determined whether the substitutes exerted their effect on sprouting via VEGF and/or uPAR.

## Results

### Histological features of skin substitutes

**Fig. 1:**
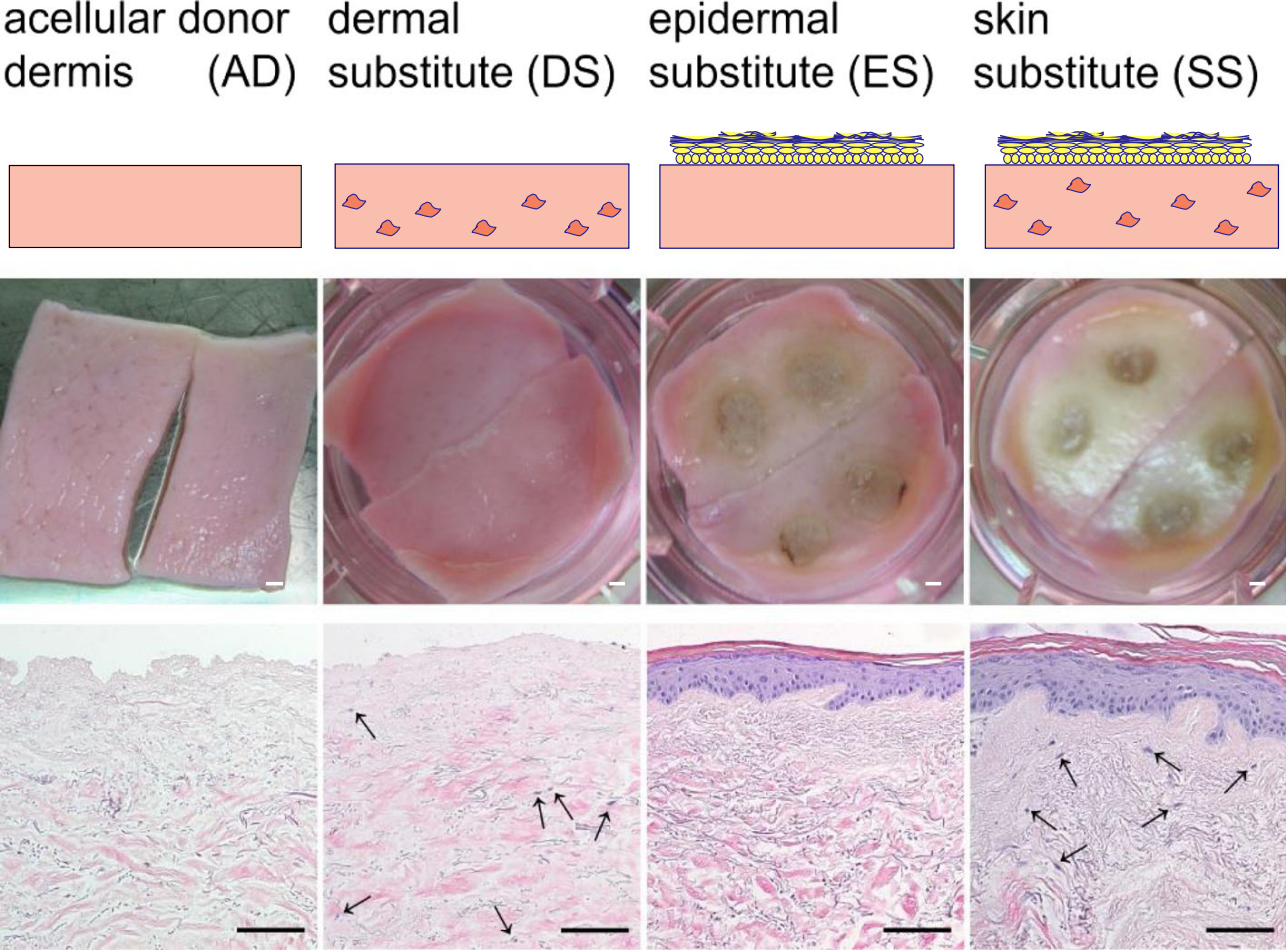
Overview of the skin substitutes. Upper panels show macroscopic view and lower panels show haematoxylin and eosin staining of tissue sections of AD, DS, ES and SS. Arrows indicate fibroblasts in the dermis. White bars represent 1000 μm and black bars 100 μm

The different skin substitutes used in this study are shown in Fig. 1. The SS consists of a fully differentiated epidermis expanding over the donor dermis which has become repopulated with fibroblasts. Each batch (transwell) of SS is derived from 2 pieces of AD and 4 × 3 mm diameter skin punch biopsies. DS is the same construct as SS but without the epidermis and ES is the same construct as SS but without the fibroblasts. The acellular donor dermis (AD) is the matrix used to construct SS, DS and ES and is used as a control throughout in the experiments described below. These constructs have been extensively described previously [12].

### Influence of skin substitute secretomes on endothelial cell proliferation and migration

**Fig. 2:**
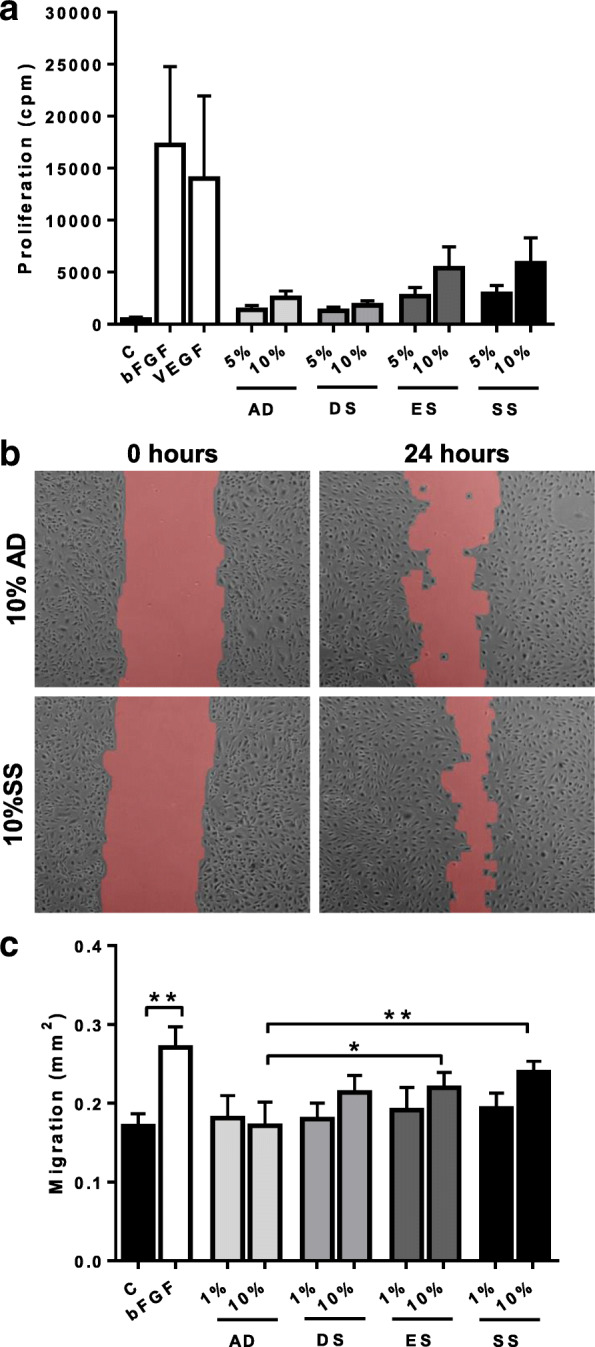
Proliferation and migration of endothelial cells in response to DS, ES, SS secretomes. **a** Proliferation: endothelial cell culture medium was supplemented with bFGF (10 ng/ml) and VEGF (10 ng/ml) or AD, DS, ES, SS secretome (5 and 10%) and ^**3**^H uptake determined 72 h later. **b** Scratch assay: representative pictures of human dermal endothelial cells cultured in the presence of 10% AD secretome or 10% SS secretome at 0 and 24 h. **c** Scratch assay: migration of endothelial cells into the scratch area in response to bFGF (10 ng/ml) or AD, DS, ES and SS secretome (1 and 10%). Area covered (mm^**2**^) by migrated endothelial cells is shown. C = unsupplemented endothelial cell cultures; significance was determined using a repeated measures one-way ANOVA followed by a Dunnett’s multiple comparisons test. *P < 0.05, **P < 0.01. Data is shown for 4–5 donors as mean ± SEM. cpm = counts per minute

In order to determine the potential of the different skin substitutes to stimulate angiogenesis, first the secretome obtained from DS, ES and SS was compared with control AD for its ability to stimulate endothelial cell proliferation and migration. The secretome consisted of the SS culture medium and soluble proteins secreted by the living skin substitutes and therefore AD secretome (SS medium not conditioned by living cells) was used as a negative control in the experiments. For proliferation, the amount of ^3^H incorporated into endothelial cells over a 72 h culture period was determined (Fig. 2a). Endothelial cells exposed to bFGF or VEGF was used as positive controls in the proliferation experiments and gave a 59.7 and 46.6 fold increase in endothelial cell proliferation respectively compared to unexposed cell cultures. Whereas strong trends were observed, significance was not reached due to donor variation between independent experiments. The acellular AD conditioned culture medium already slightly induced proliferation compared to unstimulated endothelial cells (8.8 fold) and therefore results for DS, ES and SS are expressed relative to AD. The secretome of DS did not stimulate proliferation, whereas 10% ES secretome (2.0 fold) and 10% SS secretome (2.3 fold) slightly stimulated endothelial cells to proliferate compared to AD.

Next, the ability of the DS, ES and SS secretomes to stimulate endothelial cell migration was determined using the scratch wound closure assay (Fig. 2b). This assay has been extensively described previously (22). The positive control bFGF stimulated a 1.6 fold increase in endothelial cell migration compared to the unsupplemented cultures (*P* < 0.01). The AD conditioned culture medium did not result in any increase in endothelial cell migration. When endothelial cell cultures were supplemented with 10% secretome derived from DS, ES and SS a small but significant increase in endothelial cell migration was observed for ES and SS relative to AD (DS: 1.3 fold, *P* = 0.08; ES: 1.4 fold, *P* < 0.05; SS: 1.5 fold P < 0.01).

### Sprouting of endothelial cells induced by biopsies from epidermal- and skin substitutes is largely mediated by VEGF

**Fig. 3:**
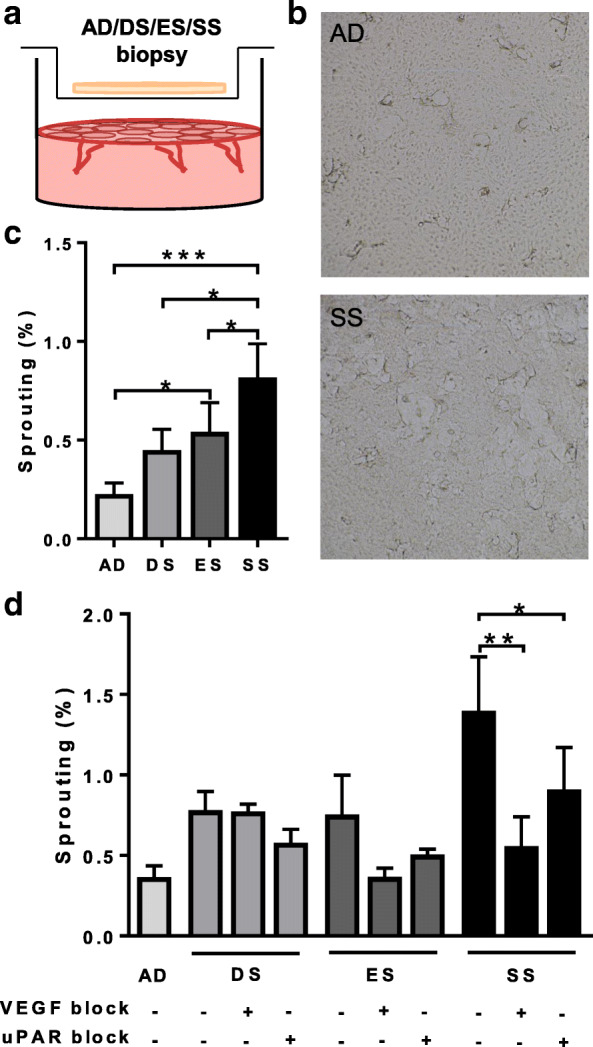
In vitro sprouting of endothelial cells into a fibrin hydrogel in response to skin substitute biopsies. **a** Schematic overview of a 6 mm biopsy (AD, DS, ES or SS) in a transwell above a 3D fibrin hydrogel with a confluent layer of EC on top. **b** Representative pictures of sprouting assay using human dermal endothelial cells. Pictures show endothelial cells on a fibrin gel exposed to SS or AD biopsy. **c** Quantification of sprouting in response to AD, DS, ES, SS biopsies after 24–48 h exposure. **d** Quantification of sprouting in response to AD, DS, ES, SS biopsies after 48–72 h (24 h longer than Fig. 3b so blocking can be observed better). Within an independent experiment, quantification of sprouting occurred at single time point for all variables and was dependent on the sprouting potential of the donor. The exposure is combined with a VEGF or uPAR blocking agent. Significance of stimulation was determined using a Friedman test followed by a Dunn’s multiple comparisons test or a repeated measures one-way ANOVA followed by a Dunnett’s multiple comparisons test. *P < 0.05. Data is shown for 4 donors as mean ± SEM

Endothelial cell sprouting involves cell proliferation, migration and degradation of the 3D matrix and plays a vital role in early angiogenesis. The sprouting assay used in this study involved a 3D fibrin hydrogel with a confluent layer of endothelial cells on top, cultured in HMEC medium (Fig. 3). In order to create a more physiologically relevant situation, mimicking skin substitute application to the wound bed, enabling crosstalk to occur between living cells (rather than a secretome), we next biopsied (6 mm diameter) the living skin substitutes and placed the biopsies in a transwell hanging above the endothelial cells on the fibrin hydrogels (Fig. 3a). Within 24–48 h the first sprout formation was evident. SS biopsies most strongly induced sprout formation compared to AD 3.6 fold, *P* < 0.001). ES biopsies were less potent than SS biopsies (2.4 fold, *P* < 0.05) and DS biopsies were least potent (2.0 fold, *P* = 0.08) (Fig. 3b).

Keratinocytes secrete large amounts of VEGF and the uPA receptor plays a key role in the ability of endothelial cells to degrade and invade the fibrin matrix [13, 14]. To investigate the induced sprout formation further, blocking experiments were performed the VEGF inhibitor Avastin and uPAR inhibitor to determine the role of VEGF and uPAR in the induced sprout formation (Fig. 3c). Induction of sprout formation by SS biopsies was almost completely inhibited by the VEGF inhibitor (*P* < 0.01) and to a lesser extent the uPAR inhibitor (*P* < 0.05). The induction of sprout formation by ES biopsies was also inhibited by the VEGF and uPAR inhibitors. The very slight induction of sprouting by DS biopsies was not blocked by the VEGF inhibitor Avastin and blocking uPAR only resulted in partial inhibition of sprout formation in 2 out of 4 donors. Taken together these results indicate that the SS has a more potent angiogenic potential than ES or DS and that VEGF and uPAR are key players regulating vessel sprouting.

## Discussion

For chronic wounds it is of great importance that granulation tissue formation and angiogenesis are stimulated to restore the disturbed wound healing process. In this study the mode of action of DS, ES and SS in the treatment of chronic ulcers was investigated. We show here that the bi-layered SS is more potent than DS or ES in inducing migration and sprouting of endothelial cells. This is in line with Wojtowicz et al. who showed that the secretome of ES and SS is more potent than DS in the maintenance of a vascular network of macrovascular endothelial cells (HUVEC) on top of Matrigel [15].

An important stimulator of endothelial proliferation, migration and sprouting is VEGF which is highly secreted by keratinocytes [14, 16, 17]. We found that ES and SS were more potent in stimulating sprouting than the DS and that this sprouting was indeed inhibited by the VEGF inhibitor Avastin. In a previous study, in which we investigated the secretome of DS, ES and SS, we found that ES and SS secrete more VEGF than DS [12]. Also another study showed that SS, and to a lesser extent ES, secreted more VEGF than DS [15]. VEGF can directly stimulate sprouting, but can also induce sprouting via induction of uPA secretion by endothelial cells, which is an important protein for matrix degradation and also for the invasion of endothelial cells into the matrix [13]. The slight induction of sprouting by DS was not mediated by VEGF, but was in part stimulated through uPAR, showing that DS only affect endothelial cell sprouting via this mechanism. Indeed fibroblasts have been reported to secrete uPA [18]. SS and ES mediated sprouting was also partly reduced when uPAR was blocked, which is in line with the finding that VEGF is highly secreted by ES and SS and in turn stimulates uPA secretion by endothelial cells [14]. This indicates that the epidermal compartment, by secreting VEGF, is mainly responsible for the induction of endothelial cell sprouting, but that synergistic interactions between the cells in the epidermis and dermis results in the most potent skin construct. To induce sprouting continuous stimulation of sprouting by skin construct biopsies was required, since stimulating with the secretomes did not induce sprout formation (data not shown). It is therefore possible that the results obtained in the proliferation and migration assays may also be greatly enhanced if living skin constructs were used to stimulate the endothelial cells rather than the secretomes.

Our data suggests that SS might stimulate granulation tissue formation by stimulating endothelial sprouting. This is in line with clinical observations that show that SS revitalize the inert chronic wound bed and induce granulation tissue formation [1]. Regarding burn wounds, excess granulation tissue formation and ECM deposition is thought to result in hypertrophic scar formation [19, 20]. For the purpose of burn wounds it might therefore be prudent to use a less potent skin construct, e.g. the ES or DS rather than the SS. Indeed in the clinic, generally only keratinocyte containing products and non-cultured skin autografts have been described to close burn wounds rather than bi-layered SS. Cultured keratinocytes have been reported to suppress excessive granulation tissue formation in the burn wound bed [21]. Of note, we have previously applied the SS to three acute surgical wounds and hypergranulation occurred in all 3 cases indicating that the SS is indeed a very potent stimulator of angiogenesis [10].

## Conclusions

Our results indicate that, during the treatment of chronic wounds with an ES or SS, the endothelial cells will be triggered to form sprouts via VEGF and activation of uPAR. This in vitro study supports our clinical observations that a bi-layered SS, containing autologous healthy fibroblasts and keratinocytes, is a strong stimulator of angiogenesis and therefore has the potential to revitalize an inert wound bed.

## Methods

### Human tissue and ethical considerations

Human skin was obtained from healthy individuals undergoing routine surgical procedures. The discarded skin was collected anonymously if patients or legal guardians, had not objected to use of their rest material (opt-out system). Foreskin from young healthy individuals after circumcision (age < 6 years) and human adult tissue from individuals undergoing abdominal dermolipectomy (age > 18 years) was used. Tissue collection procedures were in compliance with the ‘Code for Proper Secondary Use of Human tissue’ as formulated by the Dutch Federation of Medical Scientific Organization (http://www.federa.org) and with the approval of the local medical research ethics committee (MREC) of the Amsterdam UMC.

### Culture of skin substitute (SS), epidermal substitute (ES) and dermal substitute (DS)

SS, ES and DS were constructed from human foreskin as described previously (Patent International Publication No. WO 2005/068614 A2) [10, 12]. In brief, intact epidermal sheets were separated using dispase from the dermis of 4 × 3 mm diameter punch biopsies and placed on 2 pieces of acellular donor dermis (2.5 × 1.5 cm^2^). Epidermal sheets on acellular donor dermis were cultured air-exposed in SS medium (DMEM (BioWhittaker, Verviers, Belgium)/Ham’s F-12 (Invitrogen, GIBCO, Paisley, UK)(3:1), 1% penicillin/streptomycin (P/S) (Invitrogen, GIBCO, Paisley, UK), 1 μM hydrocortisone, 1 μM (−)-Isoproterenol hydrochloride, 0.1 μM insulin, 4 ng/ml keratinocyte growth factor (KGF) and 1 ng/ml epidermal growth factor (EGF) and supplemented with 1% UltroSerG (UG)(BioSepra SA, Cergy-Saint-Christophe, France). Primary fibroblasts isolated from the dermis of the same 3 mm diameter biopsies were cultured in 0.4 mm pore size transwells (Cat nr: 3450; Costar Corning Incorporated, Corning, NY) until at least 70% confluent (approximately 1 week) in DMEM containing 1% UG and 1% P/S. Next, the acellular donor dermis containing the epidermal sheet was placed onto the fibroblasts in order to allow fibroblast migration into the donor dermis and epidermis migration over the dermis and this construct is further referred to as SS. The SS was cultured at the air-liquid interface in SS medium supplemented with 0.2% UG, 10 μM l-carnitine, 10 mM l-serine, 0.4 mM L-Ascorbic acid, 1 μM dl-α-tocopherol acetate, and a lipid supplement containing 25 μM palmitic acid, 15 μM linoleic acid, 7 μM arachidonic acid and 24 μM bovine serum albumin for another 14 to 21 days until the epidermal sheet had expanded over the donor dermis. The cultures received new culture medium twice a week. Unless otherwise stated, all culture additives were obtained from Sigma-Aldrich (St. Louis, MO, USA). Culture procedures for ES were as described for SS, only fibroblasts were omitted and culture procedures for DS were as described for SS, only the epidermal sheet was omitted. As control acellular donor dermis without fibroblasts and epidermal sheets (AD) was cultured in parallel. SS, ES, DS and AD were cultured under identical conditions. Within one experiment a single foreskin donor and acellular dermis donor were used to construct SS, ES, DS and AD. All constructs were harvested at the same time for the sprouting assay (biopsy) and histological analysis. Culture supernatants (1.5 mL/culture/24 h) were collected and is referred to as secretome of SS, ES, DS or AD.

### Cell isolation and culture of endothelial cells

Dermal derived endothelial cells were isolated from human healthy adult skin as described previously [22]. Endothelial cells were cultured in pre-coated plates with 1% gelatin (Sigma-Aldrich, St. Louis, MO) in M199 (Lonza, Verviers, Belgium), 10% newborn calf serum (NBCS) (Invitrogen, Paisley, UK), 10% Human Serum (Sanquin, Netherlands), 1% P/S, 2 mM L-glutamine (Invitrogen, Paisley, UK), 5 U/ml Heparin (Leo Pharmaceutics Products, The Netherlands) and 0.0375 mg/ml endothelial cell growth factor (ECGF) (prepared from bovine brain, department of Physiology, VUmc, Amsterdam, The Netherlands) [23]. For all experiments the endothelial cells were used between passage 4 and 10.

### Proliferation assay

Proliferation of endothelial cells in response to the secretome of SS, ES, DS or AD was determined using ^3^H-thymidine incorporation, method adapted from [22]. In short, endothelial cells were seeded on 1% gelatin-coated culture plates in a density of 6 × 10^3^ cells/cm^2^ in M199 medium with 10% NBCS and 1% P/S. After 16 h, the endothelial cells were exposed for 72 h to the secretome of SS, ES, DS or AD (0, 5 and 10% v/v) or 10 ng/ml recombinant human VEGF_165_ (Preprotech, London, UK) or 10 ng/ml bFGF (Preprotech, London, UK). During the last 16 h of growth, 1 μCi ^3^H-thymidine (Perkin Elmer, Belgium) was added to quantify the amount of DNA replication as a measure for proliferation. The beta-emission was measured with Ultima Gold scintillation fluid on a 1900 TR Liquid Scintillation Analyzer (Packard Bioscience, Massachusetts, USA).

### Cell migration assay

Migration of endothelial cells in response to the secretome of SS, ES, DS or AD was determined using a scratch assay as described previously [22]. Shortly, a confluent layer of endothelial cells was cultured in M199, 10% NBCS, 10% Human Serum 1% P/S and 2 mM L-glutamine (HMEC medium) for 8 h before the start of the experiment. A scratch was drawn in the confluent monolayer with a plastic disposable pipette tip (1000 μl), after which the endothelial cells cultures were washed to remove any loose cells. Then the cells were exposed to HMEC medium supplemented with secretome of SS, ES, DS or AD (0, 1, 10%) or 10 ng/ml bFGF. Photographs of the wound area were taken at t = 0 h and t = 16 h using phase contrast microscopy. The photographs were analyzed using an image processing algorithm by which the damaged area was measured [24]. The closed area was determined by subtracting the damaged area at time point t = 16 h from t = 0 h.

### In vitro angiogenesis sprouting assay

In vitro tube formation in response to biopsies of SS, ES, DS and AD was studied using 3D fibrin matrices, using a method adapted from Koolwijk et al [25]. Briefly, fibrin matrices were prepared by addition of thrombin (0.5 U/mL) (MSD, The Netherlands) to a 3 mg/mL fibrinogen (Enzyme Research Laboratories, Leiden, The Netherlands) solution in M199 medium. Hydrogels were pipetted into a 24-well plate (400 μl). After polymerization, thrombin was inactivated by incubating the matrices with HMEC medium. Endothelial cells were seeded at a confluent density of 5.3x10^4^cells/cm^2^ onto the fibrin hydrogels. The endothelial cells in the 24-well-plate were stimulated with HMEC or HMEC supplemented with 5 μg/ml uPAR inhibitor (R&D Systems, Abingdon, UK), 200 μg/ml Avastin® (bevacizumab) (Roche, Welwyn Garden City, United Kingdom) or corresponding isotype control. After 4 h, 0.4um transwells (Cat nr: 3470; Costar Corning Incorporated, Corning, NY) containing 6 mm biopsies of SS, ES, DS and AD were placed above the endothelial cells on the fibrin hydrogels. The sprouts formed by endothelial cells into the fibrin matrices were photographed and analyzed using a Nikon Eclipse 80*i* microscope and NIS-elements AR software 3.2. The amount of sprouting is expressed as surface area of the sprouts as a percentage of the total surface of the picture.

### Histological analysis

Constructs were formalin-fixed and embedded in paraffin according to standard protocols. Paraffin embedded sections of 5 μm were stained with haematoxylin and eosin for morphological analysis. The sections were photographed using a Nikon Eclipse 80*i* microscope.

### Data analysis and statistical analysis

Statistical analyses were performed using T-tests or one-way ANOVA tests. All data is available upon request.

For collection of secretome: within an experiment, the same foreskin donor was used isolate keratinocytes and fibroblasts required to construct DS, ES and SS. Acellular dermis within an experiment was also obtained from a single donor (but a different donor to that from which cells were isolated). Secretome from 5 independent experiments was collected. These independent secretome batches were used to expose to endothelial cells. For endothelial cells: within an experiment, the same adult skin donor was used. For each independent experiment, a different donor was used. All data was obtained from four or five independent experiments with intra-experiment duplicates (sprouting) and triplicates (proliferation, migration) being performed in parallel wells. Differences were considered significant when *P < 0.05, **P < 0.01, ***P < 0.001. Results are shown as mean ± SEM.

## Data Availability

The datasets used and/or analysed during the current study are available from the corresponding author on reasonable request.

## Abbreviations

AD: Acellular donor Dermis
DS: Dermal Substitute
ES: Epidermal Substitute
SS: Skin Substitute
uPAR: urokinase Plasminogen Activator Receptor
VEGF: Vascular Endothelial Growth Factor
